# Depression, subjective cognitive decline, and the risk of neurocognitive disorders

**DOI:** 10.1186/s13195-019-0527-7

**Published:** 2019-08-09

**Authors:** Tau Ming Liew

**Affiliations:** 1 Department of Geriatric Psychiatry, Institute of Mental Health, 10 Buangkok View, Singapore, 539747 Singapore; 2 Saw Swee Hock School of Public Health, National University of Singapore, Singapore, Singapore

**Keywords:** Subjective cognitive complaints, Geriatric depression scale, Mild cognitive impairment, Dementia, Cohort study, Cox regression

## Abstract

**Background:**

Depression and subjective cognitive decline (SCD) both predict neurocognitive disorders (NCD). However, the two correlate strongly with each other. It remains uncertain whether they reflect independent neurobiological underpinnings which deserve separate attention. This study evaluated the independent risks of NCD associated with depression and SCD.

**Methods:**

This cohort study included 13,462 participants who were ≥ 50 years and had normal cognition at baseline. The participants were evaluated for depression and SCD and followed up almost annually for incident mild cognitive impairment or dementia (MCI/dementia) (median follow-up = 4.4 years). Depression and SCD were included in Cox-regression to investigate their independent risks of MCI/dementia.

**Results:**

At baseline, 1307 participants (9.7%) had depression and 3582 (26.6%) had SCD. During follow-up, 1490 (11.1%) developed MCI/dementia. Depression and SCD demonstrated independent risks of MCI/dementia (HR 1.4 and 2.0 respectively). The risk was highest when depression and SCD co-occur (HR 2.8), with half of the participants in this group developing MCI/dementia within 7.2 years of follow-up (compared to 12.2 years in participants without depression or SCD).

**Conclusions:**

The findings may change the clinical approach in managing SCD in depression, suggesting the need for greater emphasis on detecting prodromal NCD. They may also have implications to our understanding of NCD, suggesting the need for further research to delineate the commonalities and distinctions in the neurobiological pathways of depression and SCD.

**Electronic supplementary material:**

The online version of this article (10.1186/s13195-019-0527-7) contains supplementary material, which is available to authorized users.

## Introduction

Subjective cognitive decline (SCD) refers to a person’s subjective experience of worsening in cognition (typically in the memory domain), in the absence of objective cognitive deficits [1]. It is increasingly common with advancing age [2], with large community-based studies in the literature pointing to a prevalence of 50–60% among older persons [3, 4]. In recent years, SCD has gained attention as a plausible predictor for incident neurocognitive disorders (NCD) and has been suggested to be useful in the diagnosis of prodromal NCD [1, 5]. In the 2018 NIA-AA research framework for Alzheimer’s disease [6], SCD has been postulated to be a transition phase in the continuum from normal cognition to early NCD.

Notwithstanding the evidence supporting the usefulness of SCD, the literature has not been conclusive on the validity of SCD in identifying prodromal NCD [7, 8]—especially in the context of psychiatric disorders such as depression [1, 5]—and was specifically highlighted in the recently published research framework on SCD [1, 5]. SCD can often co-occur with depression among older persons [1, 5, 7, 8] and is not uncommonly the chief complaint of older persons with depression [8]. Considering such strong correlation between SCD and depression [1, 5, 7, 8], the literature remains uncertain whether SCD truly represents a separate disease process from that of depression, or is merely an alternate measure of a common neurobiology which is also related to depression [7, 8].

Using a large sample, this study sought to provide the confirmatory evidence on whether SCD truly has an independent effect—separate from that of depression—on the risk of mild cognitive impairment (MCI) and dementia, and hence reflects an independent neurobiological underpinning that deserves separate attention from that of depression.

## Methods

### Study population

This cohort study is based on the National Alzheimer’s Coordinating Center (NACC) [9] database, involving participants who were recruited from the Alzheimer’s Disease Centers across the USA and followed up almost annually for incident mild cognitive impairment (MCI) and dementia. It included participants who fulfilled the following criteria: (1) recruited between September 2005 and May 2018, (2) aged ≥ 50 years, (3) diagnosed as having normal cognition at baseline (that is, participants had received clinical evaluations at baseline and found not to have MCI or dementia), and (4) completed the Geriatric Depression Scale (GDS) and a question on SCD at baseline. Research using the NACC database was approved by the University of Washington Institutional-Review-Board. Written informed consents were obtained from all the participants.

### Measures

SCD was evaluated with a single yes/no question based on whether the participant perceived “a decline in memory relative to previously attained abilities”. The focus on the memory domain is not inconsistent with the current evidence in the literature, particularly in the recently proposed SCD framework [1], where memory concerns have been suggested to demonstrate better likelihood (than other non-memory concerns) in detecting prodromal NCD. GDS [10] assesses the level of depressive symptoms over the past week using 15 yes/no questions. The responses are summed to produce a total score, with higher scores indicating higher levels of depressive symptoms. The original GDS includes, among the 15 items, an item that seems to also capture the construct of SCD (item 10: Do you feel you have more problems with memory than most?). To avoid confounding the relationship between depression and SCD, this item 10 was excluded from the total score of GDS, resulting in a total maximum score of 14 (instead of the original total score of 15). In this study, GDS ≥ 4 was used to identify those with depression. This is not inconsistent with the findings from a recent diagnostic meta-analysis [11], where GDS ≥ 4 was identified as the optimal cut-off score to detect *major depression*—it has the highest Diagnostic Odds Ratio across the various cut-off scores, as well as demonstrated a good balance between sensitivity and specificity (88% and 86%, respectively). Notwithstanding this, alternative cut-off scores (GDS > 0, GDS ≥ 5, and GDS ≥ 6) were also tested in the subsequent sensitivity analyses to examine the robustness of the results to the choice of cut-off score. The Mini-Mental-State-Examination (MMSE) [12] was measured in this study and included in the analyses as one of the potential confounders. MMSE is a widely used cognitive assessment tool. It consists of 11 items across cognitive domains such as orientation, memory, concentration, language, and constructional praxis.

The diagnoses of MCI and dementia were made based on all available information from standardized assessments [9], with 74.1% made via consensus conference and the remainder made by single clinicians. MCI was diagnosed using the modified Petersen criteria [13], while dementia was diagnosed using either the McKhann (1984) criteria [14] or the McKhann (2011) criteria [15].

### Statistical analyses

Cox proportional-hazard regression was conducted to evaluate the risk of MCI and dementia related to depression and subjective cognitive decline, with time-to-event defined as the duration from study recruitment to the diagnosis of either MCI or dementia. In the Cox regression, the baseline presence of depression (GDS ≥ 4) and SCD was concurrently included to evaluate the unique risks that were attributable to each of them (after adjusting for the effects of each other). The cox regression adjusted for potential confounders that are known to predict neurocognitive disorders [16], including the baseline covariates of age, sex, ethnicity, years of education, family history of dementia, current smoking, hypertension, hyperlipidemia, diabetes mellitus, and MMSE.

The proportional hazard assumption of Cox regression was tested statistically based on whether the Schoenfeld residuals were associated with time—variables that violated the proportional hazard assumption (*P* < 0.05) were included in the Cox regression as stratified variable. Inverse probability weighting (IPW) [17] was used in Cox regression to account for participants who did not have follow-up data. IPW is a well-accepted strategy which gives more weight to participants who resemble those who did not have follow-up data and ensures that the results are less biased towards participants who provided follow-up data [17, 18]. As such, this method minimizes any potential bias in the results due to differential risks between those with and without follow-up data. Further details on IPW are available in Additional file 1.

Six sensitivity analyses were conducted to evaluate the consistency of the results when some parts of the main analysis were modified. They included:

1. Using the presence of *depressive symptoms* (GDS > 0), instead of the presence of *depression* (which was defined as GDS ≥ 4)
2. Using a more stringent cut-off score of GDS ≥ 5 to define depression (instead of GDS ≥ 4)
3. Using an even more stringent cut-off score of GDS ≥ 6 to define depression (instead of GDS ≥ 4)
4. Adjusting additionally for the covariate of antidepressant use at baseline (of note, 18.2% of the participants reported the use of antidepressant at baseline)
5. Using dementia as the primary endpoint (instead of the composite endpoint of mild cognitive impairment or dementia)
6. Analyzing only the complete cases with available follow-up data (*n* = 10,219)

Additionally, a stratified analysis was conducted to evaluate the risks of MCI and dementia across different combinations of presentation, as classified by the presence of depression or SCD at baseline. All statistical analyses were conducted in Stata (version 14).

## Results

**Fig. 1:**
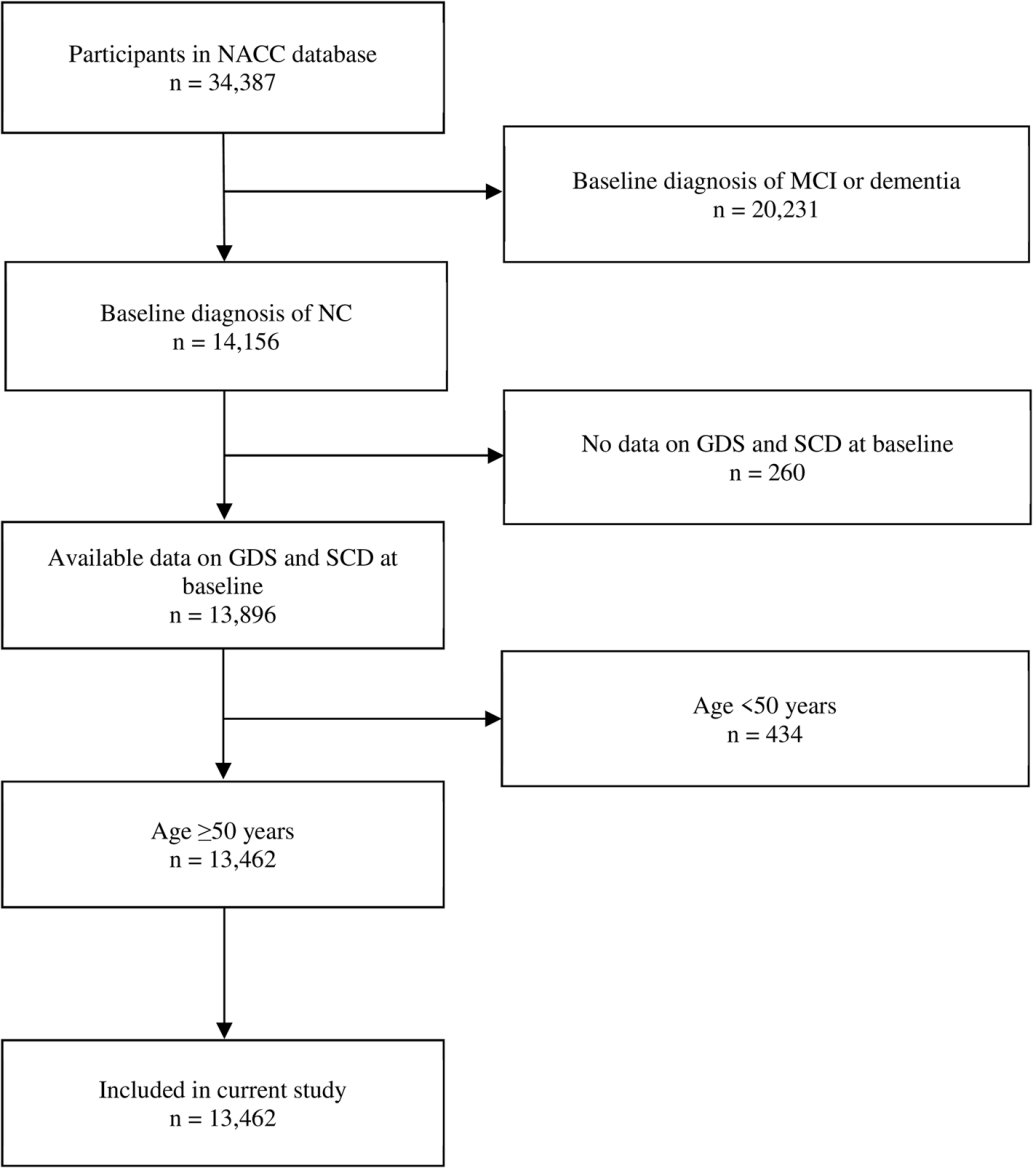
Participant enrolment and exclusion details. NACC, National Alzheimer’s Coordinating Center; MCI, mild cognitive impairment; NC, normal cognition; GDS, geriatric depression scale; SCD, subjective cognitive decline

A total of 13,462 participants were included in this study, of which 24.1% only had baseline data and did not contribute to follow-up data, while the rest of the participants had a median duration of follow-up of 4.4 years (interquartile range, IQR 2.2–7.4 years). The flow diagram related to participant selection is shown in Fig. 1, while the participant characteristics (as well as the comparison between participants with and without follow-up data) are presented in Table 1. The participants had a median age of 71 (IQR 65–78) and a median MMSE score of 29 (IQR 28–30). At baseline, 1307 participants (9.7%) reported the presence of depression (GDS ≥ 4), while 3582 (26.6%) reported the presence of SCD. During follow-up, 1490 (11.1%) converted to MCI, while 695 (5.2%) converted to dementia (with 533 being Alzheimer’s dementia, 62 vascular dementia, 34 dementia with Lewy Bodies, 13 frontotemporal lobar degeneration, and 53 due to other or unknown etiology).

**Table 1:** Demographic information of the study participants at baseline (*n* = 13,462) and comparison between those with and without longitudinal follow-up data

Variable	Overall sample (*n* = 13,462)	Participants with follow-up data (*n* = 10,219)	Participants without follow-up data (*n* = 3243)	*P* value^a^
Age, median (IQR)	71 (65–78)	72 (66–78)	70 (64–76)	**<** *0.001*
Years of education, median (IQR)	16 (14–18)	16 (14–18)	16 (14–18)	0.353
Male sex, *n* (%)	4629 (34.4)	3541 (34.7)	1088 (33.6)	0.250
Ethnicity, *n* (%)				**<** *0.001*
White	10,633 (79.0)	8209 (80.3)	2424 (74.8)	
African American	1924 (14.3)	1397 (13.7)	527 (16.3)	
Others/unknown	905 (6.7)	613 (6.0)	292 (9.0)	
Marital status, *n* (%)				*< 0.001*
Married	7988 (59.3)	6095 (59.6)	1893 (58.4)	
Widowed	2566 (19.1)	2047 (20.0)	519 (16.0)	
Divorced/separated	1964 (14.6)	1382 (13.5)	582 (18.0)	
Single	829 (6.2)	611 (6.0)	218 (6.7)	
Other/unknown	115 (0.9)	84 (0.8)	31 (1.0)	
Living arrangement, *n* (%)				*< 0.001*
Lives alone	4445 (33.0)	3409 (33.4)	1036 (32.0)	
Lives with spouse	7817 (58.1)	5970 (58.4)	1847 (57.0)	
Lives with relative or friend	933 (6.9)	675 (6.6)	258 (8.0)	
Lives with group/other	267 (2.0)	165 (1.6)	102 (3.2)	
Type of residence, *n* (%)				*< 0.001*
Private residence	12,207 (90.7)	9146 (89.5)	3061 (94.4)	
Retirement community	950 (7.1)	821 (8.0)	129 (4.0)	
Assisted living/nursing home/other	305 (2.3)	252 (2.5)	53 (1.6)	
Primary reason of participation, *n* (%)				*< 0.001*
To participate in research	11,848 (88.0)	9088 (88.9)	2760 (85.1)	
For clinical evaluation	1240 (9.2)	927 (9.1)	313 (9.7)	
For clinical evaluation and participate in research	359 (2.7)	192 (1.9)	167 (5.2)	
Unknown	15 (0.1)	12 (0.1)	3 (0.1)	
Primary source of referral, *n* (%)				*< 0.001*
Self/relative/friend	5685 (42.2)	4217 (41.3)	1468 (45.3)	
Healthcare providers	2441 (18.1)	1634 (16.0)	807 (24.9)	
Other	4924 (36.6)	4016 (39.3)	908 (28.0)	
Unknown	412 (3.1)	352 (3.4)	60 (1.9)	
Family history of dementia, *n* (%)	7274 (54.0)	5700 (55.8)	1574 (48.5)	*< 0.001*
Current smoker, *n* (%)				*< 0.001*
Yes	664 (4.9)	507 (5.0)	157 (4.8)	
No	12,774 (94.9)	9703 (95.0)	3071 (94.7)	
Missing data	24 (0.2)	9 (0.1)	15 (0.5)	
Diabetes mellitus, *n* (%)				*0.008*
Yes	1592 (11.8)	1160 (11.4)	432 (13.3)	
No	11,832 (87.9)	9028 (88.4)	2804 (86.5)	
Missing data	38 (0.3)	31 (0.3)	7 (0.2)	
Hypertension, *n* (%)				0.225
Yes	6628 (49.2)	5074 (49.7)	1554 (47.9)	
No	6795 (50.5)	5116 (50.1)	1679 (51.8)	
Missing data	39 (0.3)	29 (0.3)	10 (0.3)	
Hyperlipidemia, *n* (%)				0.452
Yes	6609 (49.1)	5038 (49.3)	1571 (48.4)	
No	6693 (49.7)	5065 (49.6)	1628 (50.2)	
Missing data	160 (1.2)	116 (1.1)	44 (1.4)	
MMSE score, median (IQR)	29 (28–30)	29 (28–30)	29 (28–30)	0.607
GDS score, median (IQR)	1 (0–2)	1 (0–2)	1 (0–2)	*< 0.001*
Presence of depression (GDS ≥ 4), *n* (%)	1471 (10.9)	993 (9.7)	478 (14.7)	*< 0.001*
Presence of SCD, *n* (%)	3582 (26.6)	2605 (25.5)	977 (30.1)	*< 0.001*

*IQR* interquartile range, *MMSE* Mini-Mental State Examination, *GDS* geriatric depression scale, *SCD* subjective cognitive decline

^a^Test of difference between participants with and without longitudinal follow-up data: chi-square test for categorical variables and Mann-Whitney U test for continuous variables. Italicized *P* values are ≤ 0.05

In Cox regression, both depression and SCD demonstrated independent risks of MCI and dementia (hazard ratio, HR of 1.4 for depression and 2.0 for SCD) (Table 2). The findings remained consistent in the six sensitivity analyses, with minimal change to the risk estimates of depression and SCD, and are further presented in Additional file 2.

**Table 2:** The risk of mild cognitive impairment and dementia based on the presence of depression and subjective cognitive decline (*n* = 13,462)

Adjustment model	Depression		SCD	
	HR (95% CI)^a^	*P* value	HR (95% CI)^a^	*P* value
Model 1 (unadjusted) ^a^	1.4 (1.2–1.6)	< 0.001	2.0 (1.8–2.2)	< 0.001
Model 2 ^b^	1.5 (1.3–1.7)	< 0.001	2.1 (1.9–2.3)	< 0.001
Model 3 ^c^	1.4 (1.2–1.6)	< 0.001	2.1 (1.9–2.3)	< 0.001
Model 4 (final) ^d^	1.4 (1.2–1.6)	< 0.001	2.0 (1.9–2.2)	< 0.001

*SCD* subjective cognitive decline, *HR* hazard ratio, *CI* confidence interval

^a^Cox-regression included only depression and SCD without covariate adjustment

^b^Cox-regression adjusted for covariates of age, sex, and ethnicity

^c^Covariate adjustment as in model 2, with additional adjustment for years of education, family history of dementia, current smoking, diabetes mellitus, hypertension, and hyperlipidemia

^d^Covariate adjustment as in model 3, with additional adjustment for Mini-Mental State Examination score

**Fig. 2:**
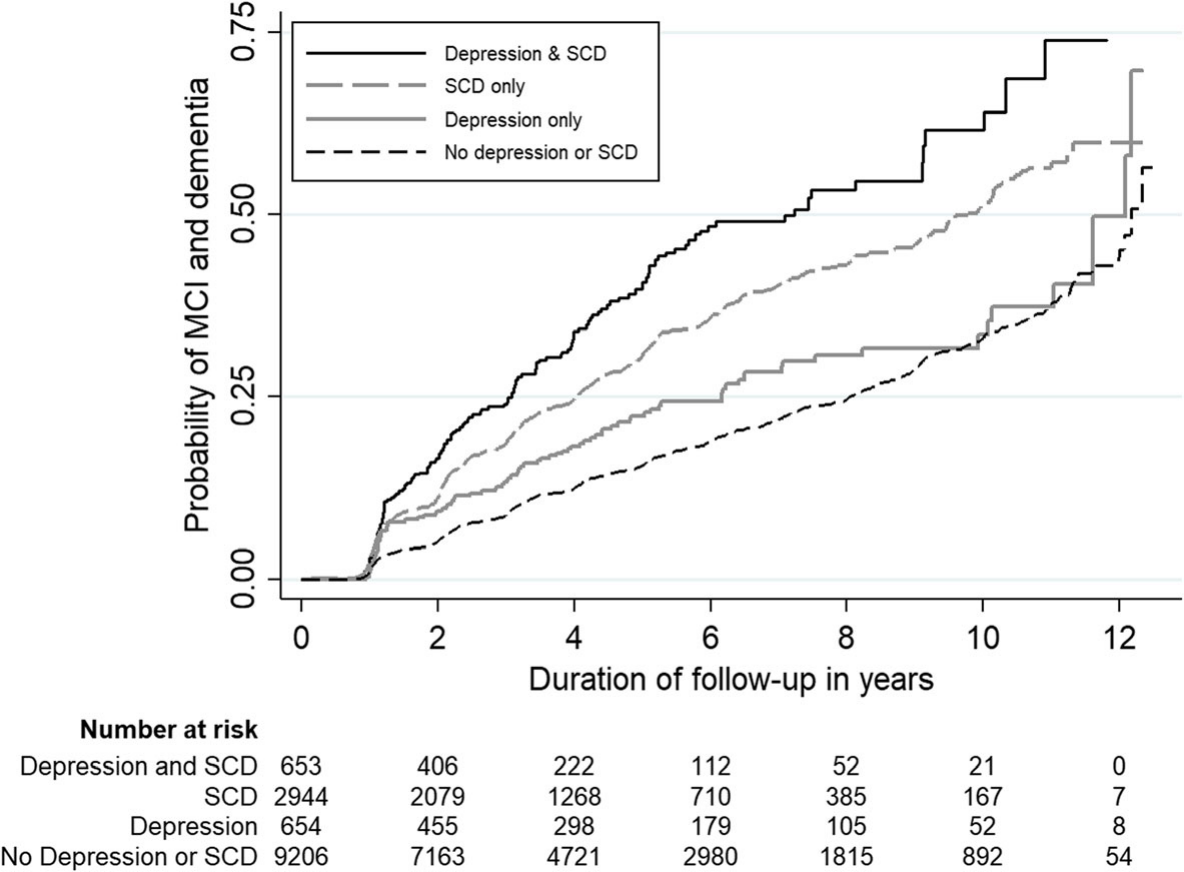
Kaplan-Meier curves reflecting the risk of mild cognitive impairment and dementia across the different combinations of presentation (*n* = 13,462). MCI, mild cognitive impairment; SCD, subjective cognitive decline

The risks of MCI and dementia were then stratified by the presence of depression or SCD at baseline. As shown in Table 3, the HR of MCI and dementia increased incrementally from depression only (HR 1.4), to SCD only (HR 2.0), and to both depression and SCD (HR 2.8). Notably, there was no overlap in the 95% CI of the different combinations of presentation, reflecting the significance of the respective increment in HR. Among individuals without depression or SCD, half of them developed MCI or dementia within 12.2 years of follow-up. This duration shortened to 7.2 years in the presence of both depression and SCD. The Kaplan-Meier curves for the different combinations of presentation are presented in Fig. 2.

**Table 3:** Risk of mild cognitive impairment and dementia across the different combinations of presentation (*n* = 13,462)

Different combinations of presentation	HR (95% CI)^a^	*P* value	Median time to MCI and dementia, year (95% CI)^b^
No depression or SCD	1.0 (Ref)	Ref	12.2 (12.1–12.3)
Depression only	1.4 (1.1–1.7)	0.004	12.1 (11.6–12.2)
SCD only	2.0 (1.8–2.2)	< 0.001	9.8 (9.1–10.2)
Both depression and SCD	2.8 (2.4–3.4)	< 0.001	7.2 (5.2–9.1)

*HR* hazard ratio, *CI* confidence interval, *MCI* mild cognitive impairment, *SCD* subjective cognitive decline, *Ref* reference group

^a^Model adjusted for baseline variables of age, sex, ethnicity, years of education, family history of dementia, current smoking, diabetes mellitus, hypertension, hyperlipidemia, and Mini-Mental State Examination score

^b^The 95% CI was computed with 1000 bootstrap sampling

## Discussion

This study utilized a large sample of cognitively normal older persons and a longitudinal study design, to investigate the relationships among depression, SCD, and incident NCD. Both depression and SCD were independently associated with the risk of MCI and dementia, with HR of 1.4 and 2.0, respectively. The results were robust to several sensitivity analyses. Co-occurring depression and SCD had the highest risk of developing NCD (HR 2.8), with half of the participants in this group developing NCD within 7.2 years of follow-up (compared to 12.2 years in participants without depression or SCD).

The findings may have implications to health services which are involved in the care of older persons. SCD often co-occurs with depression [1, 5, 7, 8] and is not uncommonly the primary presentation of older persons with depression to many health services [8]. Until recently, the evidence has been uncertain on the unique role of SCD on NCD, in the context of depression [1, 5, 7, 8]. This has translated into the prevailing practice where SCD in depression is viewed primarily as a mood-related symptom [7], with minimal emphasis to follow-up on the patients’ cognitive function or monitor for the onset of NCD (apart from the initial cognitive screening to rule out NCD as the primary diagnosis). The findings from the current study allow us to draw a more definite conclusion on the independent role of SCD in depression and may potentially change our approach in the management of SCD among older persons with depression. In older patients with depression, the presence of SCD can indicate a very high risk of NCD. While the focus on managing depression remains pertinent to improve the quality of life of the patients, there may be an equally relevant need to closely monitor these patients for incident NCD. Potentially, the newer biomarkers of NCD (such as those related to amyloid protein, tau protein, and neuronal injury) [6] may be useful in these patients to identify those at very early stages of NCD for timely preventive interventions, especially when the biomarkers become more accessible to general clinicians in the foreseeable future. In future preventive trials, the identification of co-occurring depressive symptoms and SCD may also be a useful recruitment strategy to select cognitively normal individuals who are at high risk of developing NCD [19], given that these individuals are more likely to develop NCD within a shorter time frame, and hence, the efficiency of clinical trials may be improved by reducing the duration of follow-up to the outcome of interest.

The findings may potentially also have implications to our understanding of NCD. Depression and SCD have been shown to correlate strongly with each other in prior studies [1, 5, 7, 8] and, hence traditionally, have often been understood as arising from the same construct along the continuum of depressive symptoms [7, 8]. The findings from this study suggest that depression and SCD are plausibly two distinct constructs that may independently lead to NCD, which then raises further question, on whether the two may also have distinct neurobiological pathways that lead to NCD. Prior studies have already implicated different sets of neurobiology for depression and SCD. For example, in studies of neurotransmitters, the monoaminergic system in the brain stem have been associated with depression [20], while the cholinergic system in the basal forebrain has been linked to SCD [21]. In studies of neuroanatomical regions, changes in entorhinal, anterior cingulate, and left middle frontal cortices have been associated with depression in patients with prodromal NCD [22], while white matter lesions, smaller left hippocampal volumes, and temporal lobe atrophy have been linked to SCD [23]. However, it remains uncertain whether the reported neurobiological evidences are still shared between depression and SCD, which indicates that depression and SCD are merely two presentations of a common NCD pathology, or whether depression and SCD may involve two distinct neurobiological pathways that converge to lead to NCD. Further research is needed to clarify on this uncertainty—if the latter hypothesis may plausibly be true, the delineation of differing pathways may potentially improve our understanding on the pathogenesis of NCD as well as identify new drug targets which may inform future development of disease-modifying drugs for NCD.

Several limitations should be considered. First, the participants in the study involved those who volunteered at the Alzheimer’s Disease Centers. They may be more representative of patients who voluntarily present to healthcare settings than those in the community. Second, depression in this study was defined based on established cut-off scores on GDS. Although GDS does not produce a definitive diagnosis of clinical depression, this depression scale has been shown in a recent diagnostic meta-analysis [11] to have excellent sensitivity and specificity, especially at its optimal cut-off score of GDS ≥ 4, in detecting *major depression*. Moreover, the results remained consistent in the sensitivity analyses, even with alternative cut-off scores of GDS (that is, GDS ≥ 5 and GDS ≥ 6), which lend some credence to the validity of the findings. Third, the SCD measure in this study was based on a single question and focused on the memory domain. While this may not be an uncommon practice in the current literature on SCD [24, 25], such SCD measure may not have captured the full range of memory concerns or other non-memory domains. Fourth, the diagnoses of MCI and dementia were made by single clinicians in 25.9% of the participants. They may not necessarily be as accurate as those made via consensus conference.

## Conclusion

Depression and SCD demonstrated independent risks on the subsequent development of NCD, with the risk being highest when both co-occur. The findings may change the clinical approach in the management of SCD in depression, suggesting the need for greater emphasis on detecting prodromal NCD when older patients with depression present with SCD. They may also have implications to our understanding of NCD, suggesting the need for further research to delineate the commonalities and distinctions in the neurobiological pathways of depression and SCD.

## Additional files

Additional file 1: Details on the conduct of inverse probability weighting to account for those who did not have follow-up data after the first visit. (DOCX 74 kb)

Additional file 2 Results from the six sensitivity analyses to evaluate the robustness of the main findings. (DOCX 73 kb)

## Data Availability

The data were obtained from the National Alzheimer’s Coordinating Center (NACC). For further information on access to the database, please contact NACC (contact details can be found at https://www.alz.washington.edu/WEB/researcher_home.html).

## Abbreviations

AP: Attributable proportion
CI: Confidence interval
GDS: Geriatric Depression Scale
HR: Hazard ratio
IPW: Inverse probability weighting
IQR: Inter-quartile range
MCI: Mild cognitive impairment
MMSE: Mini-Mental State Examination
NACC: National Alzheimer’s Coordinating Center
NCD: Neurocognitive disorders
SCD: Subjective cognitive decline
