# Supplementary material for: Depression, subjective cognitive decline, and the risk of neurocognitive disorders
Source: Alzheimers Res Ther. 2019 Aug 9;11:70. doi: 10.1186/s13195-019-0527-7 (PMC6689179; doi:10.1186/s13195-019-0527-7)
Supplement: Supplementary file 1 — Details on the conduct of inverse probability weighting to account for those who did not have follow-up data after the first visit. (DOCX 74 kb) [file 13195_2019_527_MOESM1_ESM.docx]

**Additional file 1.** Details on the conduct of inverse probability weighting to account for those who did not have follow-up data after the first visit.

In inverse probability weighting, the “complete cases” (those with follow-up data, n=10,219) were weighted in cox regression by the inverse of their probability of being a complete case so that the results bear more semblance to those who did not contribute to follow-up data (n=3,243). The probability of being a complete case was generated from logistic regression, with the predictors based on the variables included in the primary analysis (presence of depression, presence of subjective cognitive decline, age, sex, ethnicity, years of education, family history of dementia, current smoking, hypertension, hyperlipidemia, diabetes mellitus, and Mini-Mental State Examination score) as well as other auxiliary variables that may help to predict the presence of follow-up data (marital status, living arrangement, type of residence, primary reason of participation, and primary source of referral). This logistic model had a good fit in the Hosmer-Lemeshow test (p=0.382), with the calibration plot showing agreement between the predicted probability and the observed frequency as shown below:
