# Supplementary material for: Depression, subjective cognitive decline, and the risk of neurocognitive disorders
Source: Alzheimers Res Ther. 2019 Aug 9;11:70. doi: 10.1186/s13195-019-0527-7 (PMC6689179; doi:10.1186/s13195-019-0527-7)
Supplement: Supplementary file 2 — Results from the six sensitivity analyses to evaluate the robustness of the main findings. (DOCX 73 kb) [file 13195_2019_527_MOESM2_ESM.docx]

**Additional file 2.** Results from the six sensitivity analyses to evaluate the robustness of the main findings.

| Sensitivity analyses | Depression | |  | SCD | | |
| --- | --- | --- | --- | --- | --- | --- |
|  | HR (95% CI) ^a^ | P-value |  | HR (95% CI)  ^a^ | P-value |  |
| 1) Presence of depressive symptoms (GDS>0) at baseline | 1.2 (1.1–1.3) | <0.001 |  | 2.1 (1.9–2.3) | <0.001 |  |
| 2) More stringent definition of depression (GDS≥5) | 1.3 (1.1–1.6) | 0.001 |  | 2.1 (1.9–2.3) | <0.001 |  |
| 3) Even more stringent definition of depression (GDS≥6) | 1.4 (1.2–1.7) | 0.001 |  | 2.1 (1.9–2.3) | <0.001 |  |
| 4) Additional covariate adjustment of antidepressant use at baseline | 1.3 (1.2–1.5) | <0.001 |  | 1.9 (1.8–2.1) | <0.001 |  |
| 5) Primary endpoint of dementia | 1.6 (1.2–2.2) | 0.001 |  | 2.0 (1.7–2.5) | <0.001 |  |
| 6) Complete case analysis (n=10,219) | 1.4 (1.2–1.6) | <0.001 |  | 2.0 (1.9–2.2) | <0.001 |  |

SCD, subjective cognitive decline; HR, hazard ratio; CI, confidence interval; GDS, Geriatric Depression Scale.

^a^ The model included the presence of depression and SCD at baseline, and adjusted for baseline variables of age, sex, ethnicity, years of education, family history of dementia, current smoking, diabetes mellitus, hypertension, hyperlipidaemia and Mini-Mental State Examination score.
